# Editorial: Advanced biomaterials for hard tissue repair and regeneration

**DOI:** 10.3389/fbioe.2024.1476088

**Published:** 2024-09-25

**Authors:** Hongye Yang, Dandan Pei, Wenjie Zhang, Jian Yu

**Affiliations:** ^1^ State Key Laboratory of Oral and Maxillofacial Reconstruction and Regeneration, Key Laboratory of Oral Biomedicine Ministry of Education, Hubei Key Laboratory of Stomatology, School and Hospital of Stomatology, Wuhan University, Wuhan, China; ^2^ Key Laboratory of Shaanxi Province for Craniofacial Precision Medicine Research, College of Stomatology, Xi’an Jiaotong University, Xi’an, China; ^3^ College of Stomatology, Shanghai Jiao Tong University, National Center for Stomatology, National Clinical Research Center for Oral Diseases, Shanghai Key Laboratory of Stomatology, Shanghai Engineering Research Center of Advanced Dental Technology and Materials, Shanghai, China; ^4^ Division of Endodontics, Department of Oral Biological and Medical Sciences, Faculty of Dentistry, University of British Columbia, Vancouver, Canada

**Keywords:** bone, tooth, hard tissue, repair, regeneration, tissue engineering

Bone and tooth are typical hard tissues in vertebrates. Due to hierarchical structural characteristics and excellent mechanical properties, hard tissues play important roles for human body, such as health protection, movement support, and food mastication. Once hard tissue defect occurs, our living quality will be seriously affected. In general, hard tissues lack the ability of self-repair, except for the regeneration ability of bone for small-scale defects. As a result, the past few decades have witnessed great progress in the field of biomaterials for hard tissue repair. Actually, both bone and tooth are masterpieces of biomineralization in nature, the repair and regeneration of hard tissues should be performed in a biomimetic way. Therefore, this Research Topic provides a comprehensive overview of the current state of the art regarding advanced biomaterials used to repair or regenerate damaged hard tissues, as well as to highlight the most promising advanced strategies other than suggest the future direction in the field. In the present Research Topic, 154 authors from all over the world decided to publish their outstanding and promising results.

1. For bone regeneration, biomaterials should serve as desirable scaffolds not only to offer a microenvironment closer to *in vivo* conditions, but also to exhibit excellent bioactivity, biocompatibility, and osteogenic properties. Combined with cell seeding and bioactive molecule functionalization, a faster repair rate and better regeneration quality in bone defects would be expected.

In particular, Zhu et al. present a comprehensive review of recent developments in responsive scaffolds, thereby contributing novel insights to the field of bone defect repair.

Nelson et al. tested the efficacy of mineral coated microparticles (MCM) and fluoride-doped MCM (FMCM) to effectively deliver firefly luciferase (FLuc) mRNA lipoplexes (LPX) to the fracture site, and found that FMCM-LPX-FLuc could serve as a promising mRNA delivery platform for fracture healing applications.

Sui et al. described the advances in materials used for minimally invasive treatment of vertebral compression fractures and enumerated the types of bone cement commonly used in current practice. They also discussed the limitations of the materials themselves, and summarized the approaches for improving the characteristics of bone cement.

Zhao et al. established a model of chronic infectious mandibular defect (IMD) by mixed infection with *Staphylococcus aureus* and *Pseudomonas aeruginosa*, further explored the occurrence and development of IMD and identified key genes by transcriptome sequencing and bioinformatics analysis.

Sun et al. proposed a submicron forest-like (Fore) silicon surface based on photoetching, and found that the upregulation of macrophage M2 polarization on the Fore surface contributed to enhance osteogenesis *in vitro* and accelerate bone regeneration *in vivo*.

Wang et al. assessed the impact of low-intensity pulsed ultrasound (LIPUS) therapy on the peri-implant osteogenesis in a Type II diabetes mellitus (T2DM) rat model. The results showed that LIPUS has a great potential for T2DM patients to attain improved peri-implant osteogenesis.

Xu et al. developed Ti-24Nb-4Zr-8Sn scaffolds with methacrylated gelatin and deferoxamine, providing a new strategy to improve the osteogenesis and angiogenesis for repair of large bone defects.

Wang et al. suggested that, biomaterial scaffolds seeded with allogenic fetal BMSCs represent a promising strategy to induce and improve bone regeneration under diabetic condition.

Liu et al. constructed a novel injectable strontium-doped hydroxyapatite bone-repair material, which demonstrated good antibacterial properties, biocompatibility, and osteoinductivity.

Finally, Guo et al. reviewed the current mainstream types and characteristics of hydrogels, and summarized the relevant basic research on hydrogels in promoting periodontal tissue regeneration and bone defect repair in recent years.

2. For tooth repair, biomimetic approaches should be developed to remineralize dentin, manage root canal, enhance the restorative materials with bonding/antibacterial ability, or mimic natural teeth from multiple perspectives (such as morphology, strength, and color). Accordingly, new biomaterials, methodologies, and approaches for tooth repair are continually invented.

In particular, Zhao et al. suggested that the acetone-wet bonding (AWB) technique was effective in enhancing the dentin bond durability by increasing the wettability of dentin surface to adhesives, removing residual water in the hybrid layer, improving the penetration of adhesive monomer, and inhibiting the collagenolytic activities.

Sheng et al. investigated the antimicrobial properties of Triton, an all-in-one irrigant, on *Enterococcus faecalis* and multispecies oral biofilms in dentin canals, as well as its ability to remove the smear layer.

Zhu et al. found that color adjustment potential was dependent on resin composite type, background color, and restoration depth, so shade selection is indispensable for multi-shade resin composites. Charisma Diamond One exhibited the highest color adjustment potential and the most pronounced color shifting, contributing to simplifying the process of shade selection and improving the efficiency of clinical work.

Indurkar et al. present the synthesis of ACP with naturally occurring organic compounds (ascorbate, glutamate, and itaconate) ubiquitously found in mitochondria and vital for bone remodeling and healing. This research contributes to the expanding field of biomaterial science by bridging the gap between the biomineralization process and synthetic material.

Wang et al. demonstrated that antibiofilm peptides are effective in promoting corrosion resistance of titanium against *Streptococcus mutans*, suggesting a promising strategy to enhance the stability of dental implants by endowing them with antibiofilm and anticorrosion properties.

Chen et al. synthesized quercetin-encapsulated hollow mesoporous silica nanocomposites (Q@HMSNs), which held promise for inhibiting dentine erosion and abrasion by promoting tubule occlusion and demineralized organic matrix preservation.

Jiang et al. illustrated that proper incorporation of bioceramic micro-fillers in attachments provides an innovative approach for clear aligner therapy with reinforced antibiofilm and remineralization effects without weakening shear bonding strength.

Hu et al. reported that the bond strength between ceramic brackets and zirconia was significantly lower after thermocycling compared to that of metal brackets and zirconia. SBPM exhibited consistent and robust bond strength between ceramic/metal brackets and zirconia across various storage conditions.

Chen et al. evaluated the ability of theaflavin-3,3′-digallate (TF3)/ethanol solution to crosslink demineralized dentin collagen, resist collagenase digestion, and explore the potential mechanism.

Finally, Hu et al. proved that two antimicrobial peptides (AMPs), buCaTHL4B and Im-4, possessed remarkable antibacterial and anti-biofilm capabilities against pathogenic root canal biofilms *in vitro*, indicating their potential as promising additives to optimize the effectiveness of root canal treatment as alternative irrigants.

In summary, we have gathered an array of cutting-edge research articles that delve into the exciting realm of advanced biomaterials for hard tissue repair and regeneration, particularly focusing on the domains of bone and tooth restoration. The compilation of 20 meticulously curated contributions not only showcases the remarkable progress made in this interdisciplinary field but also offers a glimpse into the promising avenues that lie ahead.

Future research should strive to address the limitations of current biomaterials, such as their long-term stability, biocompatibility, and the potential for immune rejection. The integration of advanced manufacturing techniques, such as 3D printing and precision engineering, can further refine the design and fabrication of biomaterials, enabling the creation of customized, patient-specific solutions. Moreover, a deeper understanding of the cellular and molecular mechanisms underlying hard tissue repair and regeneration is crucial for the rational design of next-generation biomaterials.

